# *Slc4a11* disruption causes duct cell loss and impairs NaCl reabsorption in female mouse submandibular glands

**DOI:** 10.14814/phy2.14232

**Published:** 2019-12-12

**Authors:** Ning‐Yan Yang, Taro Mukaibo, Xin Gao, Ira Kurtz, James E. Melvin

**Affiliations:** ^1^ Secretory Mechanisms and Dysfunctions Section National Institute of Dental and Craniofacial Research National Institutes of Health Bethesda Maryland; ^2^ Department of Pediatric Dentistry Beijing Stomatological Hospital & School of Stomatology Capital Medical University Beijing China; ^3^ Division of Oral Reconstruction and Rehabilitation Kyushu Dental University Kitakyushu Fukuoka Japan; ^4^ Department of Medicine Division of Nephrology David Geffen School of Medicine, and the Brain Research Institute University of California Los Angeles California

**Keywords:** Acinar cells, ductal cells, fluid secretion, NaCl reabsorption

## Abstract

Slc4a11, a member of the Slc4 HCO_3_^−^ transporter family, has a wide tissue distribution. In mouse salivary glands, the expression of Slc4a11 mRNA was more than eightfold greater than the other nine members of the *Slc4* gene family. The Slc4a11 protein displayed a diffuse subcellular distribution in both the acinar and duct cells of mouse submandibular glands (SMG). *Slc4a11* disruption induced a significant increase in the Na^+^ and Cl^−^ concentrations of stimulated SMG saliva, whereas it did not affect the fluid secretion rate in response to either *β*‐adrenergic or cholinergic receptor stimulation. Heterologous expressed mouse Slc4a11 acted as a H^+^/OH^−^ transporter that was uncoupled of Na^+^ or Cl^−^ movement, and this activity was blocked by ethyl‐isopropyl amiloride (EIPA) but not 4,4′‐Diisothiocyanato‐2,2′‐stilbenedisulfonic acid (DIDS). *Slc4a11* disruption revealed that Slc4a11 does not play a major role in intracellular pH regulation in mouse salivary gland cells. In contrast, NaCl reabsorption was impaired in the SMG saliva of female compared to male *Slc4a11* null mice, which correlated with the loss of duct cells and a decrease in expression of the duct‐cell‐specific transcription factor Ascl3. Together, our results suggest that Slc4a11 expression regulates the number of ducts cells in the mouse SMG and consequently NaCl reabsorption.

## Graphical abstract

Slc4a11, a member of the Slc4 HCO_3_^−^ transporter family, is highly expressed in mouse salivary glands. Slc4a11 disruption induced a significant increase in the Na^+^ and Cl^−^ concentrations of stimulated saliva, whereas it did not affect the fluid secretion rate. The NaCl reabsorption defect in female Slc4a11 null mice correlated with the loss of duct cells and a decrease in expression of the duct cell‐specific transcription factor Ascl3.

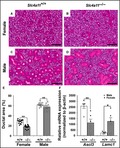

## Introduction

SLC4A11 (also known as BTR1) is a member of the *SLC4* gene family of HCO_3_^−^ transporters (Romero et al. 2013; Parker et al. 2013). SLC4A11 has the least sequence similarity to the other SLC4 HCO_3_^−^ transporter members and its function remains controversial (Parker et al. 2001). Originally thought to be an electrogenic sodium borate cotransporter (Na^+^(n)‐B(OH^−^)^4−^) or cation (Na^+^ or H^+^) permeation pathway (Park et al. 2004), subsequent studies reported that SLC4A11 mediates Na^+^–OH^−^ cotransport (equivalent to a Na^+^/H^+^ exchanger) (Jalimarada et al. 2013; Ogando et al. 2013); NH_4_^+^ permeation (Ogando et al. 2013), water flux (Vilas et al. 2013; Soumittra et al. 2014), H^+^(OH^−^) transport (Jalimarada et al. 2013; Kao et al. 2015, 2016; Myers et al. 2016), and/or NH_3_–2H^+^ cotransport (Kao et al. 2016; Zhang et al. 2015). Unlike other SLC4 transporters, SLC4A11 does not transport HCO_3_^−^ (Jalimarada et al. 2013; Ogando et al. 2013; Loganathan et al. 2016).

SLC4A11 is predominantly expressed in the kidney, salivary glands, testis, thyroid glands, trachea (Parker et al. 2001), pancreas, liver, and spleen (Park et al. 2004), as well as the cornea (Damkier et al. 2007). Mutations in SLC4A11 are considered responsible for human corneal disorders, such as autosomal recessive congenital hereditary endothelial dystrophy (CHED2) (Jiao et al. 2007; Hand et al. 2017), Harboyan syndrome (Desir and Abramowicz 2008; Liskova et al. 2015; Siddiqui et al. 2014) and Fuchs endothelial corneal dystrophy (Vithana et al. 2008; Kim et al. 2015). Similarly, *Slc4a11* disruption in mice caused corneal endothelial dystrophy (Gröger et al. 2010; Han et al. 2013) and sensorineural abnormalities (Lopez et al. 2009). Importantly, research using *Slc4a11* null mice also demonstrated that Slc4a11 plays a critical role in sodium‐mediated fluid transport in both the cornea and the kidney (Groger et al. 2010; Han et al. 2013). Moreover, in the rat submandibular salivary gland (SMG), Slc4a11 was targeted to the basolateral membrane of both acinar and duct cells, but much higher expression was observed in acinar cells (Park et al. 2004). However, little is known about the role of Slc4a11 in SMG fluid and electrolyte secretion.

The aim of this study was to address the possible roles and underlying functions of Slc4a11 in the mouse SMG. Our results demonstrate that mouse Slc4a11 mediates an EIPA‐sensitive H^+^–OH^−^ transport that is not coupled with Na^+^ or Cl^−^. We found that Slc4a11, the most abundantly expressed Slc4 family member in salivary glands, regulates NaCl reabsorption, but plays a minor role in intracellular pH regulation and does not contribute to fluid secretion in the mouse SMG. Loss of Slc4a11 in the SMG of female mice was associated with reduced *Ascl3* expression and a developmental decrease in the number of duct cells that correlated with a defect in NaCl reabsorption.

## Materials and Methods

### Animals

The *Slc4a11^−/−^* mice (C57BL/6J background) were generated and genotyped as described previously (Lopez et al. 2009). Age‐ and sex‐matched C57BL/6J mice (Jackson Laboratory) were used as wild‐type controls. Mice were housed in microisolator cages in a pathogen‐free facility with ad libitum access to laboratory chow and water with a 12‐h light/dark cycle. Experiments were performed on 10‐ to 14‐week‐old mice. All animal procedures were approved by the Animal Care and Use Committee of the National Institute of Dental and Craniofacial Research, National Institutes of Health (ASP 16‐802).

### RNA‐sequencing analyses

Gene expression analyses were performed using publicly available RNA‐sequencing data of the major mouse salivary glands, as has been previously described (Gao et al. 2018). Briefly, SMG from 6 adult C57BL/6J mice (3 male and 3 female) were surgically removed and total RNA was extracted from each sample, followed by cDNA synthesis. Illumina libraries were made from qualified fragmented cDNA and assessed by Illumina HiSeq 2500 sequencing. The data were analyzed using the platform provided by DNAnexus (Mountain View, CA) and outputted as fragments per kilobase of transcript per million of mapped reads (FPKM) values.

### Histology and immunofluorescence

Sections of paraformaldehyde‐fixed mouse submandibular glands were stained with hematoxylin–eosin (HE), and images from at least five fields per section were captured with a DS‐Fit1 Nikon camera (Nikon Corp., Japan) mounted on a Nikon Eclipse 50i microscope with a Leica Aperio CS2 20× objective (Leica Biosystems, Wetzlar, Germany). The acinar cross‐sectional area was calculated using ImageJ software (https://imagej.nih.gov/ij/), as previously described (Mukaibo et al. 2018). Additional sections were deparaffinized and underwent antigen retrieval in Tris‐EDTA‐buffered saline (pH 9.0) for 15 min, after which they were blocked in 1% BSA in PBS for 60 min at RT. Next, sections were incubated with rabbit anti‐Slc4a11 (1:100 dilution; Lopez et al. 2009) and goat anti‐Slc12a2 (NKCCl, 1:100 dilution; Santa Cruz Biotechnology, Dallas, TX) antibodies at 4°C overnight, followed by a 60 min incubation with donkey anti‐rabbit Alexa Fluor 647 (1:200 dilution; Invitrogen, Carlsbad, CA) and donkey anti‐goat Alexa Fluor 555 (1:400 dilution; Invitrogen) secondary antibodies, respectively, at RT. Slides were mounted using Fluoroshield^™^ with DAPI (Sigma‐Aldrich, St. Louis, MO) and images were captured with a confocal Olympus Fluoview microscope (Olympus America, Center Valley, PA).

### Ex vivo mouse submandibular gland perfusion

Ex vivo submandibular gland experiments were performed as reported previously (Peña‐Münzenmayer et al. 2015). Briefly, mice were anesthetized by intraperitoneal (i.p.) injection of chloral hydrate (400 mg/kg body weight) and then the SMG was removed and transferred to a temperature‐controlled perfusion chamber and perfused at 37°C with a HCO_3_^−^‐containing physiological solution. Salivation was induced with either the cholinergic receptor agonist carbachol (0.3 *μ*mol/L CCh) plus the *β*‐adrenergic receptor agonist isoproterenol (1.0 *μ*mol/L IPR) or with CCh alone. The duct was inserted into a capillary tube and SMG fluid secretion was recorded at 1‐minute intervals. The collected saliva was stored at −20°C until further analysis. The composition of the HCO_3_^−^‐containing physiological perfusion solution was (in mmol/L): 4.3 KCl, 120 NaCl, 25 NaHCO_3_, 5 glucose, 10 HEPES, 1 CaCl_2_, and 1 MgCl_2_ at pH 7.4 with continuous 95% O_2_ and 5% CO_2_ gassing.

### Ion concentration calculation

The concentration of sodium and potassium were measured by atomic absorption spectroscopy (Perkin Elmer Life Sciences 3030 spectrophotometer, Boston, MA). The chloride concentration was analyzed using an EA940 digital expandable ion analyzer (Orion Research, Jacksonville, FL). The saliva HCO_3_^−^ concentration was assayed using a Carbon Dioxide Enzymatic Assay kit (DZ122A‐K; Diazyme Laboratories, Poway, CA) according to the manufacturer’s instructions.

### CHO‐K1 cell culture and transfections

CHO‐K1 cells (Sigma‐Aldrich) were cultured and maintained as previously described (Park et al. 2001). Plasmids encoding mouse *Slc4a11* (GenBank accession no. NM_ 001081162), human *CD8A* (GenBank accession no. NM_001768) and the empty pCMV6 entry vector as a transfection control were obtained from OriGene (OriGene Technologies, Rockville, MD). Cells were grown in 10 cm diameter dishes and electroporated (Nucleofector II; Amaxa, Gaithersburg, MD) when 80–90% confluent with targeted plasmids (6 *µ*g each per reaction) using a Nucleofector kit V (Lonza, Alpharetta, GA) according to the manufacturer’s instructions and then seeded onto 5‐mm‐diameter coverslips (Warner Instrument, Hamden, CT). Intracellular pH (pH_i_) was monitored 18–20 h after electroporation.

### Submandibular gland acinar and duct cell isolation

After mice were euthanized by CO_2_ asphyxiation and cervical dislocation, the SMG was removed and finely minced. The minced tissue was digested for 20 min at 37°C in an Eagle’s minimum essential medium (MEM; Corning, Corning, NY) solution containing 1% BSA (Sigma‐Aldrich) and 0.125 mg/mL Liberase™ TL (Roche, Indianapolis, IN) with continuous 95% O_2_ and 5% CO_2_ gassing. The cell pellet was passed through a 250‐*μ*m Pierce™ Tissue Strainer (Thermo Fisher, Waltham, MA) and resuspended in MEM. Under these isolation conditions, intact ducts appear under the microscope as elongated linear structures and are easily differentiated from the spherical acini.

### Intracellular pH measurement

Transfected CHO‐K1 cells were loaded with pH‐sensitive dye by exposure to 2 *μ*mol/L of the pH indicator BCECF‐AM (2’,7‐bis‐(2‐carboxyethyl)‐5‐(and‐6)‐carboxyfluorescein, acetoxymethyl ester) and Dynabeads™ CD8 beads (1:500 v/v; Thermo Fisher) for 30 min at 37°C. The intracellular pH (pH_i_) was monitored in CD8‐positive cells decorated with beads after switching the solution from pH 7.4–6, then back to 7.4 and finally to 9. The compositions of solutions used are listed in Table 1. Either ethyl‐isopropyl amiloride (EIPA; 10, 200, 500 *μ*mol/L) or 500 *μ*mol/L of 4,4'‐Diisothiocyanato‐2,2'‐stilbenedisulfonic acid (DIDS) was added to test their effects on Slc4a11 transport activity. All solutions used were bicarbonate free except for the solutions used in Figure 5A. The method used to study Na^+^–HCO_3_^−^ cotransporter (NBC)‐like activity (Fig. 5A) was as described previously (Yang et al. 2019). Briefly, intracellular pH was monitored while switching the solution from Na^+^‐free to Na^+^‐containing media. SMG acinar and duct cells were incubated with 4 *μ*mol/L BCECF‐AM for 30 min and the pH_i_ was measured following the same protocols as those used in the CHO‐K1 experiments, but with 10 *μ*mol/L EIPA added to all solutions to inhibit Na^+^/H^+^ exchange activity. Images were captured with an inverted microscope (Olympus IX71) equipped with an OptoScan monochromator system (Cairn Research, Faversham, UK) coupled to a high‐speed digital camera (C11440; Hamamatsu, Hamamatsu City, Japan). BCECF fluorescence was excited alternately at 490 and 440 nm and emissions detected at 530 nm using Imaging Work Bench 6.0 software (IND EC BioSystems, Los Altos, CA). The osmolality of solutions was measured by a freezing‐point depression osmometer (Advanced Instruments, Norwood, MA) and adjusted to 300 mOsm/kg with sucrose. Solutions were kept at 37°C using a CL‐100 bipolar temperature controller (Warner Instruments, Hamden, CT) and gassed with 100% O_2_ (HCO_3_^−^ free) or with 95% O_2_ and 5% CO_2_ (HCO_3_^−^ containing). Intracellular pH is expressed as relative “*F*/*F*_o_” values from the 490:440 ratio of the BCECF fluorescence measurements.

**Table 1:** Solutions used in experiments to measure intracellular pH (in mmol/L).

Solutions	Na^+^	NMDG^−^	Cl^−^	K^+^	Ca^2+^	Mg^2+^	Gluconate^−^	Glucose	HEPES	Bis‐Tris	Bicine	pH
pH_e_ = 6*	145		128.3	4.3	1	1	25	5			10	6
pH_e_ = 7.4	145		128.3	4.3	1	1	25	5	10			7.4
pH_e_ = 9	145		128.3	4.3	1	1	25	5		10		9
pH_e_ = 6, Na^+^‐free		145	128.3	4.3	1	1	25	5			10	6
pH_e_ = 7.4, Na^+^‐free		145	128.3	4.3	1	1	25	5	10			7.4
pH_e_ = 9, Na^+^‐free		145	128.3	4.3	1	1	25	5		10		9
pH_e_ = 6, Cl^−^‐free	145			4.3	1	1	153.3	5			10	6
pH_e_ = 7.4, Cl^−^‐free	145			4.3	1	1	153.3	5	10			7.4
pH_e_ = 9, Cl^−^‐free	145			4.3	1	1	153.3	5		10		9

* pH_e_ = extracellular pH.

### Real‐time quantitative polymerase chain reaction (qPCR)

qPCR was performed as previously described (Mukaibo et al. 2018). Briefly, submandibular glands were removed and immediately immersed in liquid nitrogen and shipped to MyOmicsDx (Towson, MD). qPCR was performed to determine the mRNA levels for members of the ENaC/Degenerin superfamily (*Scnn1a, b,* and *g*), Na^+^/H^+^ exchangers (*Slc9a1, a2,* and *a3*), water channels (*Aqp5* and *Aqp11*), the cystic fibrosis transmembrane conductance regulator anion channel (*Cftr*), and 16 salivary gland morphology‐related genes using a Bio‐Rad CFX96 Touch™ Real‐Time PCR Detection System. mRNA levels were normalized to the expression of *β*‐actin. The specific qPCR primer pairs used are listed in Table 2 along with references associated with the rationale for gene selection.

**Table 2:** Real‐time PCR primers.

Gene	Forward	Reverse	Accession no.	Reference No
*Slc9a1*	CTTTGAGGAATTTGCCAGCTATG	GAGGTAAATCGGGAGGTGAAAG	NM_001358455	Park et al. (2001)
*Slc9a2*	ACCAACCCAAGTCCAGTATTG	GCTTCCTTATTTTCTCTTCACGG	NM_001033289	
*Slc9a3*	TCCCTCTATGGTGTCTTCCTC	CCAAACAGCAAGAAATCCAGG	NM_001081060	
*Scnn1a*	CCCCATTCTGCCTCCATAC	GGTCTTGCTCCTTGAAATTGTT	NM_011324	Catalán et al. (2010)
*Scnn1b*	CGCCTATCTTCTACCCTGATTATG	ATAGTCCTCCTGACCGATGT	NM_001272023	
*Scnn1g*	GAGAACGAGAAGGGAAAGGC	GTCAGAGGTGTCATTTGAGCA	NM_011326	
*Cftr*	GCCAAGGCAAGTCCTTCATCA	GCCATTTACCTTGGCATAGGC	NM_021050	Catalán et al. (2010)
*Aqp5*	GGTAACCTGGCCGTCAATG	GTGAAGTAGATCCCCACAAGATG	NM_009701.4	Aure et al. (2011) and Larsen et al. (2011)
*Aqp11*	CTTCCATGGCTGCATAACAATC	TTTGGAGGGTGCTCAGATTG	NM_175105.3	Larsen et al. (2010)
*Ascl3*	CCGGCTCTGAGAGTCATTTG	ATGAAGGTTGGTATTGGGAGG	NM_020051.1	Arany et al. (2011) and Yoshida et al. (2001)
*Cdh1*	AGAGAAGCCATTGCCAAGTAC	AACGAATCCCTCAAAGACCG	NM_009864.3	Walker et al. (2008)
*E2f1*	TGTTCACTCCCTCTTCCTATCT	CATAACTCCCTACCCACCATTG	NM_001291105.1	Satoh et al. (2013)
*Eda*	GGCCCTCCTGAATTTCTTCTT	CATCTGCTCCTTCACCACTTT	NM_001177937	Mukaibo et al. (2018)
*Edar*	AGGTTATCAGATATGCAGGCG	TGTTCCTGGGTCTGTTTTCC	NM_010100	
*Edar2*	GGGAAGTCTGCTAGTGGTATTT	GCAACGAATCTCTGCATTGAC	NM_001161432	
*Edaradd*	ACTCATCTGTCCTCTCCTGG	CCGGTGTCTTGAATGGGATAC	NM_133643	
*Gjb1*	GCTCACCAACAGCACATAGA	GCACCTTGTGTCTCTTTACCT	NM_001302496.1	Ihara et al. (2000)
*Itga3*	CTGGTGCCTACAACTGGAAA	CTGCACCGTGTACCCAATATAA	NM_001306071.1	Menko et al. (2001)
*Itgb1*	CAGTCCCAAGTGCCATGAG	AGTAAGCGTCCATGTCTTCAC	NM_010578.2	
*Lama1*	CCATGCCGATTTAGCCAATG	AGCGGTCACATTTATCTCCAG	NM_008480.2	Hoffman et al. (1996)
*Lamb1*	GAGAAGGGAATGAACTACACGG	GTCCAGCGATTTACAGTAGGG	NM_008482.2	
*Lamc1*	GGCTTGTTACCGACTTGTGA	CCTCCCTTTCTGCTTCCTTAAG	NM_010683.2	
*P2ry1*	TGTCTCATCAGTGGTAGCTTAAC	TCCCTCCAGCCAAACAATAC	NM_001282016.1	Schrader et al. (2005)
*Tgfb2*	GGCTTTCATTTGGCTTGAGATG	CTTCGGGTGAGACCACAAATAG	NM_001329107.1	Hoffman et al. (1996)
*Tgfb3*	CAGGATCTAGGCTGGAAATGG	GGGTTCAGGGTGTTGTATAGTC	NM_009368.3	
*β‐actin*	ACCTTCTACAATGAGCTGCG	CTGGATGGCTACGTACATGG	NM_007393	–

### Statistical analysis

Data are presented as the means ± SEM. Statistical significance was determined using the Student’s *t* test for two group comparisons and one‐way ANOVA followed by Bonferroni’s post hoc test for multiple comparisons. A *P*‐value of <0.05 was considered statistically significant. Origin 8.0 software was used for statistical calculations (Origin Lab, Northampton, MA).

## Results

### Slc4a11 is the most abundant Slc4 family member in the mouse SMG

**Figure 1:**
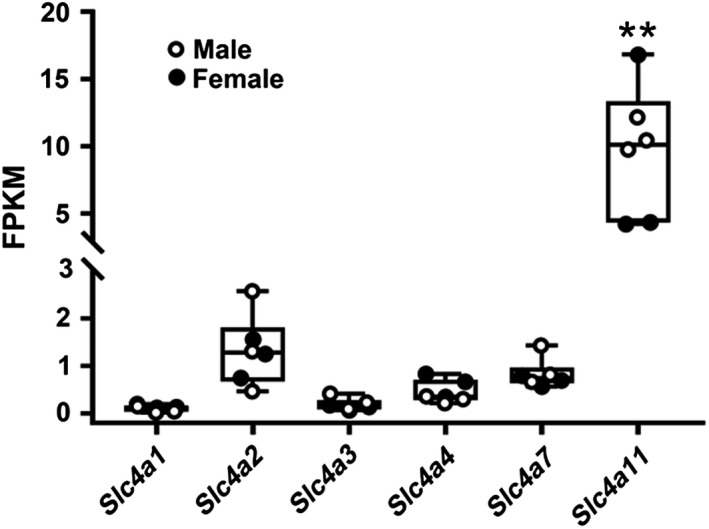
mRNA expression of *Slc4a* gene family members in the mouse SMG. The mRNA expression levels of all 10 *Slc4a* gene family members were determined from previously reported RNA‐Seq data (Gao et al. 2018). *Slc4a11* mRNA expression was at least eightfold higher (**P < 0.01) than the other *Slc4*a family members (*Slc4a1, a2, a3, a4,* and *a7*) in the mouse submandibular gland (SMG). The expression of *Slc4a11* was essentially identical between the male and female SMGs (3 male and 3 female mice; white circles = males, black circles = females). The FPKM (fragments per kilobase of transcript per million of mapped reads) values for *Slc4a5, a8, a9,* and *a10* are not shown in the figure due to their expression being less than the 0.1 cutoff. Values are shown for individual mice (circles) along with the means ± SEM. Note the break in the *Y*‐axis.

Transcriptional expression of the 10 *Slc4* family members (*Slc4a1‐5, 7‐11*) was analyzed using RNA‐Seq data previously deposited in the Gene Expression Omnibus (GEO accession number GSE96747) (Gao et al. 2018). Comparative analyses (Fig. 1) showed that *Slc4a11* mRNA expression in the mouse SMG was significantly greater (> eightfold higher) than the expression of the other *Slc4* family members *Slc4a1, a2, a3, a4,* and *a7* (*Slc4a11* FPKM = 9.63 ± 1.97, **P < 0.01). Note that the expression of *Slc4a11* was comparable in the male and female SMG (white circles = males, black circles = females) and to the mRNA expression of other ion transporters and channels that are known to be important for salivary gland function, such as *Kcnma1 (maxi‐K)*, *Ano1, Slc12a2 (Nkcc1),* and *Cftr *(4.47, 11.88, 69.6, 2.76 FPKM values, respectively, for SMG) (Mukaibo et al. 2018). Thus, *Slc4a11* is the most abundantly expressed *Slc4* family member in the mouse SMG, as well as in the mouse parotid and sublingual glands (data not shown) (Gao et al. 2018). The FPKM values for *Slc4a5, a8, a9,* and *a10* were less than the 0.1 cutoff and thus were not considered in this analysis.

### Slc4a11 is expressed in the acinar and duct cells of the mouse SMG

**Figure 2:**
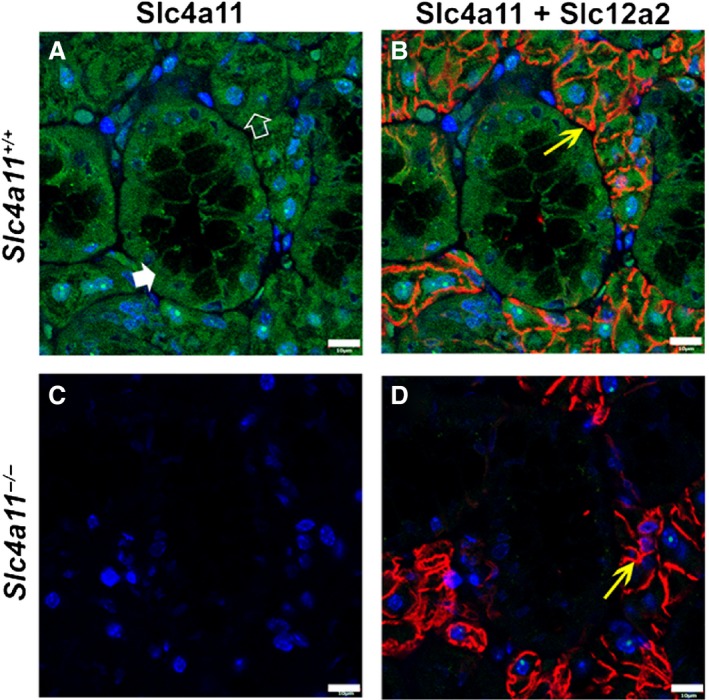
Immunolocalization of Slc4a11 in the mouse SMG. (A) Slc4a11 (green staining) expression in acinar (white open arrowhead) and duct cells (white filled arrowhead) of SMG in *Slc4a11^+/+^* mice. (B) Merged image of Slc4a11 and basolateral Slc12a2 (red staining and yellow arrow, respectively) staining in *Slc4a11^+/+^* mice. (C) Slc4a11‐specific immunostaining was not detected in the SMG of the *Slc4a11* ^−/−^ mice. (D) Merged image of Slc4a11 and Slc12a2 (yellow arrow) staining from *Slc4a11* ^−/−^ mice. Images shown are from male *Slc4a11^+/+^* and *Slc4a11* ^−/−^ mice. Scale bar = 10 *μ*m; nuclei were stained with DAPI (blue).

Slc4a11 has been detected in the basolateral membrane of rat SMG acinar and duct cells (Park et al. 2004). To better understand the function of Slc4a11 in mouse salivary glands, the cellular distribution of Slc4a11 was determined by immunofluorescence. Slc4a11‐specific staining was diffusely detected in both the SMG acinar and duct cells of *Slc4a11^+/+^* mice (Figs. 2A and 2B, green stain; filled white arrowhead = duct, open white arrowhead = acinus). Note the absence of green staining in the apical halves of the male granular duct cells (a similar pattern was observed in female granular duct cells where the granules are much smaller, not shown), consistent with the absence of Slc4a11 expression in the large secretory granules of this cell type (Pinkstaff 1980). In contrast, Slc4a11 staining was not detected in SMG acinar or duct cells of *Slc4a11* ^−/−^ mice (Figs. 2C and 2D), confirming the specificity of the Slc4a11 antibody. Immunostaining of the Na^+^–K^+^–Cl^−^ cotransporter Slc12a2 (Nkcc1) specifically labeled the basolateral membrane of acinar cells (red stain, yellow arrows), the localization and intensity of which did not appear to change in the SMG of *Slc4a11* ^−/−^ mice (Figs. 2 B and 2D). 

### *Slc4a11* disruption does not affect stimulated fluid secretion by the SMG

**Figure 3:**
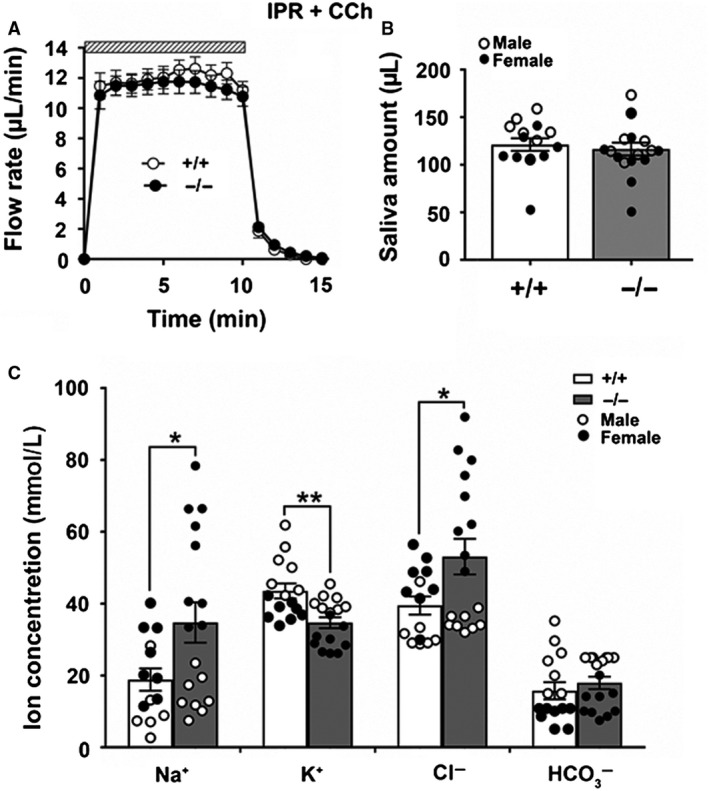
Saliva secretion and ion concentrations of the ex vivo SMG under *β*‐adrenergic and cholinergic stimulation. Mouse SMG was ex vivo perfused for 10 min with the *β*‐adrenergic receptor agonist IPR (1.0 *μ*mol/L) and the cholinergic receptor agonist CCh (0.3 *μ*mol/L) in a HCO_3_^−^‐containing physiological perfusion solution. (A) The kinetics and rates of salivation (*μ*L/min) were not significantly different between *Slc4a11^+/+^* (white circles, *n* = 15) and *Slc4a11* ^−/−^ mice (black circles, *n* = 17). (B) The saliva amounts (*μ*L) of the ex vivo SMG for *Slc4a11^+/+^* (white bar, *n* = 15, 7 males and 8 females; white circles = males, black circles = females) and *Slc4a11* ^−/−^ mice (gray bars, *n* = 17, 8 males and 9 females; white circles = males, black circles = females). (C) The Na^+^ and Cl^−^ concentrations in saliva from *Slc4a11* ^−/−^ mice (gray bars, *n* = 17, 8 males and 9 females; white circles = males, black circles = females) than *Slc4a11^+/+^* mice (white bar, *n* = 15, 7 males and 8 females; white circles = males, black circles = females) (*P < 0.01). The saliva K^+^ concentrations decreased (**P < 0.05) in the *Slc4a11* ^−/−^ mice. Data shown for individual glands (circles) along with the means ± SEM.

Given the high expression of *Slc4a11* compared to all other *Slc4a* members in the SMG and that Slc4a11 was detected in both acinar and duct cells, we predicted that Slc4a11 might play a significant role in stimulated fluid and/or electrolyte secretion. Consequently, the kinetics (time of secretion activation and deactivation upon addition and removal of agonists from the perfusate) and the amount of fluid secretion were examined in response to stimulation using the *β*‐adrenergic receptor agonist isoproterenol (IPR, sympathetic stimulation) combined with the cholinergic receptor agonist carbachol (CCh, parasympathetic stimulation) to recapitulate physiological‐like conditions (Proctor 2016). As shown in Figure 3A, ex vivo perfusion simultaneously with IPR and CCh did not significantly affect the flow rate kinetics of the SMG in *Slc4a11^+/+^* compared to *Slc4a11* ^−/−^ mice; moreover, the total amount of saliva secreted in response to CCh plus IPR was essentially identical (Fig. 3B).

### Slc4a11 disruption alters the ion composition of saliva in the mouse SMG

Saliva is generated through a two‐step process in which the acinar cells secrete a NaCl‐rich fluid and then the ducts subsequently reabsorb much of the NaCl while secreting KHCO_3_ in a *β*‐adrenergic‐dependent fashion (Denniss et al. 1978). The above results suggested that Slc4a11 was not involved in fluid secretion by mouse SMG acinar cells (Figs. 3A and 3B) but does not rule out the possibility that the expression of Slc4a11 in the ducts might contribute to the final ion composition of the saliva. Figure 3C shows that the Na^+^ and Cl^−^ concentrations significantly increased (approximately 80% and 25%, respectively, *P < 0.05) in the SMG saliva of *Slc4a11* ^−/−^ mice. In contrast, the K^+^ concentration was significantly lower in the *Slc4a11* ^−/−^ mice (approximately 15%, **P < 0.01), whereas the HCO_3_^−^ concentration and pH were essentially unchanged (pH values are summarized in Table 3). Of note, the changes in Na^+^ and Cl^−^ concentrations during IPR and CCh stimulation were restricted to female *Slc4a11* ^−/−^ mouse saliva (i.e., the Na^+^ and Cl^−^ concentrations were comparable in the saliva of male *Slc4a11^+/+^* and *Slc4a11* ^−/−^ mice; white circles = males, black circles = females). In contrast, a decreased K^+^ concentration was observed in both male and female *Slc4a11* ^−/−^ mice. Taken together, *Slc4a11* disruption altered the Na^+^ and Cl^−^ secretion in a sex‐specific manner, whereas the effect on the K^+^ concentration appeared to be independent of sex. The above results are summarized in Table 3 by sex.

**Table 3:** Characterization of submandibular salivary glands in female and male *Slc4a11^+/+^* and *Slc4a11* ^−/−^ mice.

		Female				Male			
		+/+	*n*	^−/−^	*n*	+/+	*n*	^−/−^	*n*
Body weight (g)	CCh†	19.87 ± 0.11	4	18.52 ± 0.41	5	25.06 ± 0.24	4	28.86 ± 0.30	4
	IPR + CCh‡	20.55 ± 0.23	8	18.43 ± 0.42	9	27.92 ± 0.56	7	25.28 ± 0.50	8
Gland weight (mg)	CCh	33.43 ± 0.86	4	32.60 ± 2.45	5	57.25 ± 3.64	4	55.13 ± 2.55	4
	IPR + CCh	38.20 ± 1.40	8	38.89 ± 4.11	9	69.06 ± 4.26	7	63.15 ± 0.68	8
Na^+^ concentration (mmol/L)	CCh	62.98 ± 2.25	4	**89.83** ± **3.32** **	5	30.32 ± 5.78	4	**66.97** ± **4.76** **	4
	IPR + CCh	24.63 ± 3.64	8	**52.95** ± **5.47** **	9	11.29 ± 3.67	7	14.40 ± 1.87	8
K^+^ concentration (mmol/L)	CCh	41.66 ± 5.38	4	41.82 ± 1.50	5	63.72 ± 4.56	4	**45.75** ± **3.42** *	4
	IPR + CCh	37.80 ± 0.90	8	**29.72** ± **1.21** **	9	50.21 ± 2.66	7	**40.43** ± **0.92** **	8
Cl^−^ concentration (mmol/L)	CCh	77.19 ± 5.26	4	**98.64** ± **4.13** *	5	66.04 ± 2.92	4	**81.92** ± **4.81** *	4
	IPR + CCh	45.64 ± 2.87	8	**69.36** ± **4.79** **	9	32.60 ± 2.36	7	34.86 ± 0.79	8
HCO_3_^−^ concentration (mmol/L)	CCh	5.39 ± 0.95	4	**14.56** ± **0.61** **	5	9.56 ± 0.96	4	**16.22** ± **0.56** **	4
	IPR + CCh	8.96 ± 0.90	8	12.10 ± 1.33	9	23.62 ± 2.85	7	24.64 ± 0.26	8
pH	CCh	7.43 ± 0.03	4	**7.66** ± **0.02** **	5	7.96 ± 0.06	4	8.02 ± 0.10	4
	IPR + CCh	8.90 ± 0.21	8	8.89 ± 0.19	9	9.08 ± 0.22	7	9.76 ± 0.24	8

Values are the means ± SEM, *n* represents the number of glands used in each experiment. Superscripts: † = 10 min perfusion with cholinergic receptor agonist carbachol (CCh; 0.3 *μ*mol/L), ‡ = 10 min stimulation with 1.0 *μ*mol/L IPR plus 0.3 *μ*mol/L CCh. *P < 0.05 or **P < 0.01 in bold; female *Slc4a11^+/+^* versus female *Slc4a11* ^−/−^ mice or male *Slc4a11^+/+^* versus male *Slc4a11* ^−/−^ mice.

**Figure 4:**
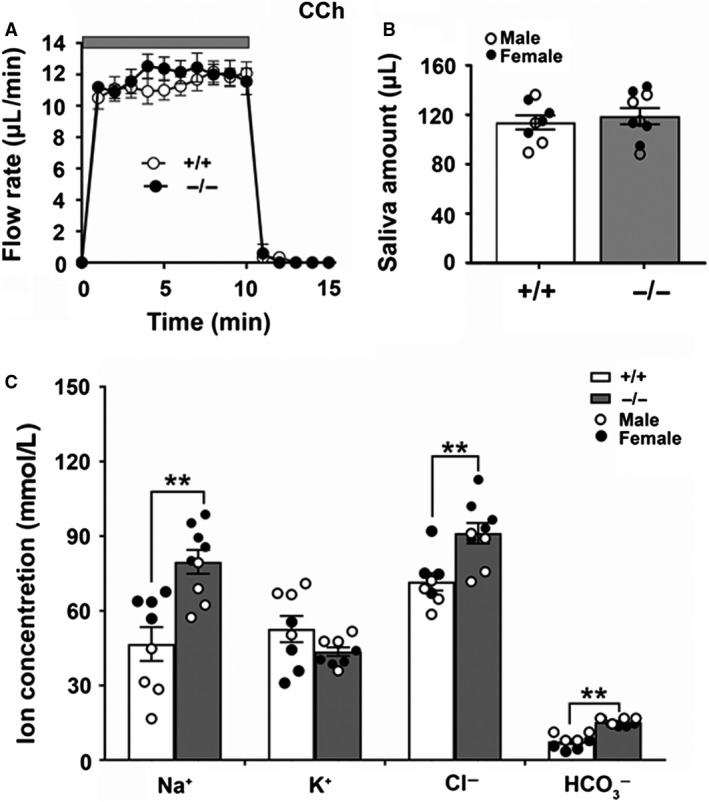
Saliva secretion and ion concentrations of the ex vivo SMG under cholinergic stimulation. Mouse ex vivo SMG was perfused for 10 min with the cholinergic receptor agonist CCh (0.3 *μ*mol/L) in a HCO_3_^−^‐containing physiological perfusion solution. (A) The kinetics and rates of salivation (*μ*L/min) in glands from *Slc4a11^+/+^* (white circles, *n* = 8) and *Slc4a11* ^−/−^ (black circles, *n* = 9) mice. (B) The saliva amounts (*μ*L) of the ex vivo SMG for *Slc4a11^+/+^* (white bar, *n* = 8, 4 males and 4 females; white circles = males, black circles = females) and *Slc4a11* ^−/−^ mice (gray bars, *n* = 9, 4 males and 5 females; white circles = males, black circles = females). (C) The Na^+^ and Cl^−^ concentrations in saliva of *Slc4a11* ^−/−^ mice (gray bars, *n* = 9, 4 males and 5 females; white circles = males, black circles = females) compared to *Slc4a11^+/+^* mice (white bars, *n* = 8, 4 males and 4 females; white circles = males, black circles = females). The saliva HCO_3_^−^ concentrations increased in the *Slc4a11* ^−/−^ mice. Data shown for individual glands (circles) along with the means ± SEM (**P < 0.01).

Given that NaCl reabsorption and KHCO_3_ secretion are likely linked to *β*‐adrenergic receptor stimulation (Denniss et al. 1978) we postulated that the sex‐specific differences in NaCl‐dependent secretion may require *β*‐adrenergic receptor stimulation. Thus, we performed ex vivo perfusion with only the cholinergic receptor agonist CCh to eliminate the involvement of *β*‐adrenergic receptor stimulation to fluid and electrolyte secretion. Similar to physiological‐like stimulation, the flow rate kinetics and total amount of saliva stimulated with CCh only were comparable in *Slc4a11^+/+^* and *Slc4a11* ^−/− ^mice (Figs. 4A and 4B). Importantly, increases in Na^+^ and Cl^−^ concentrations were still found in the saliva of *Slc4a11* ^−/−^ mice (Fig. 4C, **P < 0.01), whereas K^+^ secretion was not affected in the CCh only‐stimulated saliva. In contrast to the CCh plus IPR stimulation, a subtle but significant increase in HCO_3_^−^ was observed in the saliva of *Slc4a11* ^−/−^ mice with CCh only stimulation (Fig. 4C, **P < 0.01). These results suggested that the sex‐specific changes in NaCl reabsorption in female *Slc4a11* ^−/−^ mice were independent of *β*‐adrenergic receptor activation; thus, another mechanism must be involved (see below). Of note, the increases in Na^+^ and Cl^−^ concentrations during CCh stimulation were observed in the saliva of both female and male *Slc4a11* ^−/−^ mice. The above results are summarized in Table 3 by sex.

### ***Slc4a11 mediates EIPA‐sensitive, DIDS‐resistant, Na^+^*** ^−^ ***and Cl*** ^−^ ***‐independent H^+^‐OH^−^ transport in CHO‐K1 cells***

**Figure 5:**
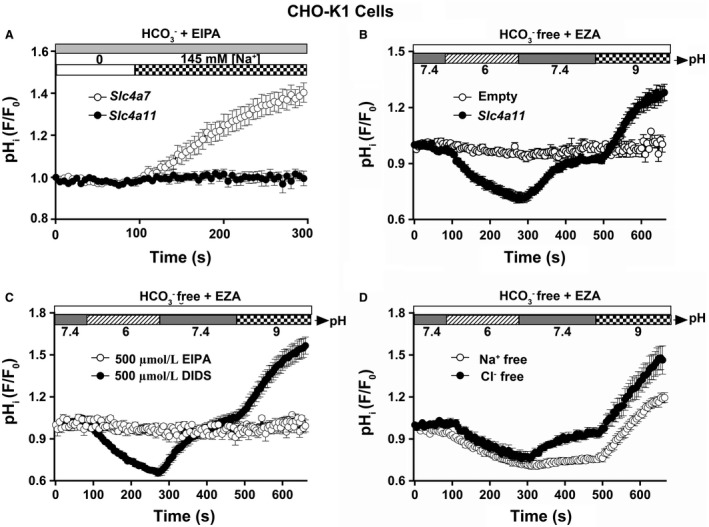
Mouse Slc4a11 mediates H^+^‐OH^−^ transport in CHO‐K1 cells. The full‐length mouse *Slc4a11* cDNA or empty vector was cotransfected with human *CD8A* cDNA, the pH‐sensitive dye BCECF was loaded, and the intracellular pH was monitored. All solutions used were bicarbonate free [in the presence of 30 *μ*mol/L carbonic anhydrase inhibitor ethoxzolamide (EZA)] except for the solutions used Figure 5A. (A) The NBC‐like activity experiment was monitored as previous described by Yang et al. (2019). A Na^+^‐dependent intracellular alkalization (HCO_3_^−^ influx) was observed in *Slc4a7*‐transfected CHO‐K1 cells (white circles, *n* = 10; note that data for *Slc4a7*‐transfected cells were transformed from our previous study (Yang et al. 2019)). In contrast, alkalization was absent in *Slc4a11*‐transfected CHO‐K1 cells (black circles, *n* = 9). (B) A significant acidification was observed in *Slc4a11*‐transfected CHO‐K1 cells when switching the cells from pH 7.4 to 6, whereas an equally remarkable alkalization was observed when switching the cells from pH 7.4 to 9 (black circles, *n* = 16). There was no significant pH change in the empty vector‐transfected CHO‐K1 cells (white circles, *n* = 12). (C) Effects of 500 *μ*mol/L EIPA or 500 *μ*mol/L DIDS on Slc4a11 activity. The Slc4a11‐mediated intracellular acidification and alkalization were completely inhibited in the presence of 500 *μ*mol/L EIPA (white circles, *n* = 10), but Slc4a11 was insensitive to 500 *µ*mol/L DIDS (black circles, *n* = 12). (D) Na^+^‐free and Cl^−^‐free solutions to address whether the Slc4a11‐mediated H^+^/OH^−^ transport activity is dependent on Na^+^ or Cl^−^. Neither the acidification nor the alkalization rate was altered in the absence of extracellular Na^+^ or Cl^−^ [white circles (*n* = 11) and black circles (*n* = 8), respectively]. Values are shown for the average results along with the means ± SEM from at least eight different individual transfections. Intracellular pH is expressed as relative “*F*/*F*_o_” values from the 490:440 ratio of the BCECF fluorescence measurements.

The above results suggest that Slc4a11 may be involved in NaCl reabsorption. Moreover, recent studies suggest that human SLC4A11 is not involved in HCO_3_^−^ transport but appears to transport H^+^(OH^−^) in a sodium‐independent fashion (Kao et al. 2015; 2016; Myers et al. 2016) although some authors have reported H^+^(OH^−^) flux is coupled with Na^+^ (Jalimarada et al. 2013; Ogando et al. 2013). Given that the ion transport characteristics of Slc4a11 have not been fully elucidated and remain controversial (Park et al. 2004; Praetorius et al. 2004; Ogando et al. 2013; Kao et al. 2015; Zhang et al. 2015), we evaluated the function of heterologous expressed mouse *Slc4a11* and *Slc4a7* in CHO‐K1 cells. Figure 5A shows that CHO‐K1 cells loaded with the pH‐sensitive fluorescent dye BCECF and expressing the mouse Na^+^‐HCO_3_^−^ cotransporter Slc4a7, also known as Nbcn1 and NBC3, mediated a Na^+^‐dependent intracellular alkalization (HCO_3_^−^ influx) when a large inward‐directed Na^+^ gradient was introduced to the Na^+^‐depleted CHO‐K1 cells [white circles; the *Slc4a7*‐transfected cell data are comparable to a previous study (Yang et al. 2019)]. In contrast, mouse Slc4a11 exhibited no Na^+^‐HCO_3_^−^ cotransporter‐like activity (Fig. 5A, black circles). Consequently, it is not surprising that the HCO_3_^−^ concentration was unchanged in the saliva of *Slc4a11* ^−/−^ null mice. Because the large change in the inward‐directed Na^+^ gradient failed to affect the pH_i_, the results shown in Figure 5A confirm that mouse Slc4a11 does not mediate Na^+^‐dependent HCO_3_^−^ transport nor does Slc4a11 likely mediate Na^+^‐coupled H^+^ and/or OH^−^ transport.

To further characterize the H^+^/OH^−^ transport activity of mouse Slc4a11, the intracellular pH was monitored in response to changes in extracellular pH in *Slc4a11*‐transfected CHO‐K1 cells in HCO_3_^−^‐free solutions (in the presence of the carbonic anhydrase inhibitor ethoxzolamide, 30 *μ*mol/L EZA) to prevent the HCO_3_^−^ transport activity that may be native to CHO‐K1 cells. There were no significant pH changes in empty vector‐transfected CHO‐K1 cells (Fig. 5B*,* white circles) when the pH of the extracellular bath was changed. In contrast, as shown in Figure 5B*, Slc4a11*‐transfected cells displayed a dramatic acidification when the extracellular bath was switched from pH 7.4 to 6, whereas an equally remarkable alkalization was observed when the bath was switched from pH 7.4 to 9 (Fig. 5B, black circles), consistent with H^+^/OH^−^ transport activity. Most Slc4 members are sensitive to DIDS (4,4'‐Diisothiocyanato‐2,2'‐stilbenedisulfonic acid) (Romero et al. 2013; Parker et al. 2013), whereas Ogando et al. (2013) reported that human SLC4A11 is an EIPA‐sensitive, Na^+^ coupled pH_i_ regulator. Figure 5C shows that the Slc4a11‐mediated H^+^/OH^−^ transport activity was completely blocked by 500 *μ*mol/L EIPA but was insensitive to 500 *μ*mol/L DIDS (white and black circles, respectively). Of note, 10 *μ*mol/L EIPA had no inhibitory effect on the H^+^/OH^−^ transport activity, whereas 200 *μ*mol/L EIPA resulted in partial inhibition (data summarized in Table 4). Na^+^‐free and Cl^−^‐free solutions were then used to address whether the H^+^/OH^−^ transport activity is dependent on Na^+^ or Cl^−^. Neither the acidification nor the alkalization was significantly affected in the absence of extracellular Na^+^ or Cl^−^ (Fig. 5D, white and black circles, respectively), suggesting that the Slc4a11‐mediated H^+^/OH^−^ transport activity is essentially independent of both extracellular Na^+^ and Cl^−^. A summary of the acidification and alkalization rates for the data shown in Figures 5B–5D are given in Table 4. These results revealed that Slc4a11 mediates H^+^/OH^−^ transport activity that does not require Na^+^ or Cl^−^ and is EIPA‐sensitive, but resistant to inhibition by DIDS. However, Table 4 shows that the empty vector‐transfected CHO‐K1 cells acidified somewhat faster under Na^+^‐free conditions than in the presence of Na^+^ whereas Slc4a11‐transfected CHO‐K1 cells acidified somewhat slower under Na^+^‐free conditions than in the presence of Na^+^. Taken together, these latter results suggest that the Slc4a11‐mediated acidification, but not alkalization, was reduced about 50% under Na^+^‐free conditions. The mechanism for the 50% reduction in the Slc4a11‐mediated acidification is not clear but is not likely due to uninhibited Na^+^/H^+^ exchange activity given that this experiment was under Na^+^‐free conditions. Alternatively, disruption of *Slc4a11* expression might alter expression or activity of other unknown pH regulatory proteins.

**Table 4:** Intracellular pH acidification and alkalization rates in empty vector‐ and *Slc4a11*‐transfected CHO‐K1 cells.

Solutions	Inhibitors	Acidification rate (10^−3^/sec, pH 7.4–6)				Alkalization rate (10^−3^/sec, pH 7.4–9)			
		Empty vector	*n*	*Slc4a11*	*n*	Empty vector	*n*	*Slc4a11*	*n*
HCO_3_^−^‐free		0.40 ± 0.16	12	1.99 ± 0.27**	16	0.16 ± 0.14	12	3.22 ± 0.40**	16
	10 *μ*mol/L EIPA	0.20 ± 0.11	5	2.66 ± 0.20**	15	0.08 ± 0.04	5	2.79 ± 0.21**	15
	200 *μ*mol/L EIPA	0.40 ± 0.13	7	1.13 ± 0.14	18	0.22 ± 0.21	7	2.26 ± 0.20**	18
	500 *μ*mol/L EIPA	0.48 ± 0.14	5	0.48 ± 0.10	10	0.04 ± 0.04	5	0.46 ± 0.09	10
	500 *μ*mol/L DIDS	0.43 ± 0.11	4	2.50 ± 0.17**	12	0.10 ± 0.09	4	3.78 ± 0.33**	12
HCO_3_^−^‐ & Na^+^‐free		0.68 ± 0.10	14	1.45 ± 0.19**	11	0.10 ± 0.03	14	3.08 ± 0.49**	11
HCO_3_^−^‐ & Cl^−^‐free		0.28 ± 0.10	6	1.73 ± 0.26**	8	0.27 ± 0.14	6	3.60 ± 0.39**	8

Values are the means ± SEM, *n* represents the number of individual transfections in each experiment.

** *P* < 0.01, empty vector‐ versus *Slc4a11‐*transfected CHO‐K1 cells.

### Slc4a11 disruption affects intracellular pH regulation in mouse SMG cells

**Figure 6:**
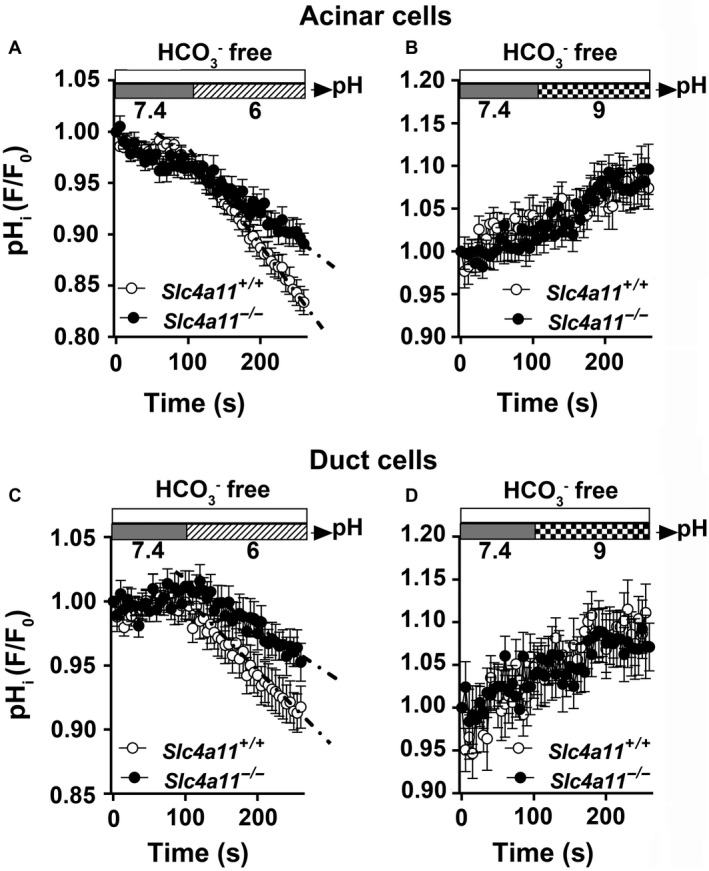
*Slc4a11* disruption has little effect on pH_i_ regulation in SMG cells. Acinar and duct cells isolated from *Slc4a11^+/+^* and *Slc4a11* ^−/−^ mouse SMGs were loaded with the pH‐sensitive dye BCECF and the intracellular pH (pH_i_) change was monitored during the switch from pH 7.4–6 or from pH 7.4–9. All solutions were HCO_3_^−^‐free (in the presence of 30 *μ*mol/L EZA) to eliminate HCO_3_^−^ transport pathways and contained 10 *μ*mol/L EIPA to block Na^+^/H^+^ exchange. (A) The acidification rate of the acinar cells isolated from *Slc4a11* ^−/−^ mice was compared to *Slc4a11^+/+^* mice (acidification rate (10^−3^/sec) of acinar cells = 0.81 ± 0.07 (*n* = 27, white circles) and 0.48 ± 0.07 (*n* = 31, black circles) for *Slc4a11^+/+^* and *Slc4a11* ^−/−^, respectively; *P* < 0.01). (B) The alkalization rate was comparable between acinar cells from *Slc4a11^+/+^* and *Slc4a11* ^−/−^ mice (alkalization rate (10^−3^/sec) of acinar cells = 0.56 ± 0.10 (*n* = 22, white circles) and 0.60 ± 0.10 (*n* = 27, black circles) for *Slc4a11^+/+^* and *Slc4a11* ^−/−^, respectively; *P* > 0.05). (C) The acidification rate of the duct cells isolated from *Slc4a11* ^−/−^ mice was compared to *Slc4a11^+/+^* mice [acidification rate (10^−3^/sec) of duct cells = 0.60 ± 0.06 (n = 24, white circles) and 0.36 ± 0.05 (n = 29, black circles) for *Slc4a11^+/+^* and *Slc4a11* ^−/−^ mice, respectively; *P* < 0.01]. (D) The alkalization rate of duct cells from *Slc4a11* ^−/−^ mice was identical to *Slc4a11^+/+^* mice [alkalization rate (10^−3^/sec) of duct cells = 0.70 ± 0.17 (*n* = 16, white circles) and 0.50 ± 0.12 (*n* = 26, black circles) for *Slc4a11^+/+^* and *Slc4a11* ^−/−^, respectively; *P* > 0.5]. Values are shown for individual SMGs along with the means ± SEM of glands isolated from 8 *Slc4a11^+/+^* and 9 *Slc4a11* ^−/−^ mice. Intracellular pH is expressed as relative “*F*/*F*_o_” values from the 490:440 ratio of the BCECF fluorescence measurements.

In Figure 5, we show that mouse Slc4a11 supports H^+^/OH^−^ transport, which could explain the previous observations that mouse SMG cells express a pH_i_ regulatory pathway that is EIPA‐sensitive and DIDS‐resistant (Luo et al. 2001; Yang et al. 2019). Taking advantage of the *Slc4a11* null mouse we examined the role of Slc4a11 in the regulation of pH_i_ in the acinar and duct cells of the mouse SMG in HCO_3_^−^‐free, EZA‐containing solutions, as above. We found that 10 *μ*mol/L EIPA did not affect the Slc4a11‐mediated H^+^/OH^−^ transport activity in CHO‐K1 cells (Table 4), thus all solutions used in the SMG experiments included 10 *μ*mol/L EIPA to block the endogenous Na^+^–H^+^ exchanger activity. Figure 6 shows that the acidification rate decreased modestly in both the acinar and duct cells of *Slc4a11* ^−/−^ mice (Figs. 6A and 6C, respectively), whereas the alkalization rate was not different (Figs. 6B and 6D, respectively). Taken together, it appears Slc4a11 plays only a minor role, if any, in the intracellular pH regulation of SMG cells.

### Slc4a11 disruption decreases the cross‐sectional area of ducts in mouse SMG

**Figure 7:**
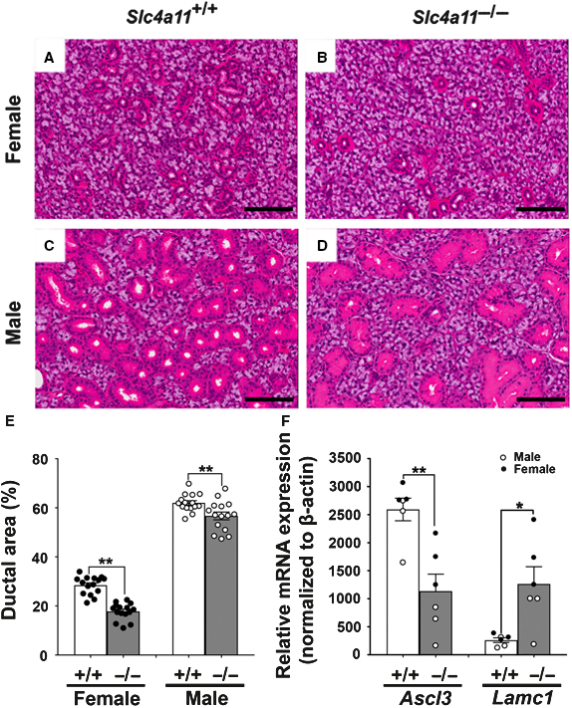
*Slc4a11* disruption alters the cross‐sectional area of duct cells in mouse SMG. Sections of paraformaldehyde‐fixed mouse SMG were stained with hematoxylin–eosin (HE). (A) female *Slc4a11^+/+^* SMG, (B) female *Slc4a11* ^−/−^ SMG, (C) male *Slc4a11^+/+^* SMG, and (D) male *Slc4a11* ^−/−^ SMG. Scale bar = 100 *μ*m. (E) The cross‐sectional areas of acinar and ductal regions were calculated as described in Materials and Methods and expressed as the % ductal area in both male and female *Slc4a11* ^−/−^ mice (gray bar, white circles = males, black circles = females) compared to *Slc4a11^+/+^* mice (white bar). Female *Slc4a11* ^−/−^ and *Slc4a11* ^+/+^ = 17.77 ± 0.89 and 28.46 ± 0.96, respectively; **P < 0.01. Male *Slc4a11* ^−/−^ and *Slc4a11* ^+/+^ = 56.71 ± 1.58 and 62.0 ± 0.92, respectively; **P < 0.01. Ductal area (%) values are shown for individual fields from sections along with the means ± SEM. (F) qPCR results for *Ascl3* and *Lamc1* expression normalized to *β‐actin* expression. The *Ascl3* and *Lamc1* mRNA expression in the SMG from *Slc4a11* ^−/−^ mice (gray bar) compared to *Slc4a11^+/+^* mice (white bar); *P < 0.05 and **P < 0.01, respectively. Values are shown for individual SMG (male = white circles, female = black circles; 3 for each) along with the means ± SEM of glands isolated from 6 *Slc4a11^+/+^* and 6 *Slc4a11* ^−/−^ mice.

The above results show that the Slc4a11 H^+^/OH^−^ transport mechanism is not dependent on Na^+^ or Cl^−^ (Fig. 5), suggesting that Slc4a11 is not directly involved in the observed changes in the NaCl composition of the SMG saliva from *Slc4a11* ^−/−^ mice (Figs. 3 and 4). Given that NaCl reabsorption primarily occurs in the duct cells (Catalán et al. 2010) we tested whether disruption of *Slc4a11* might impact the distribution of acinar and duct cells in the adult SMG. Gross morphological examination of HE‐stained SMG tissue sections revealed that the percentage of duct cells may have been altered by *Slc4a11* disruption (Figs. 7A–7D). Indeed, quantification of the cross‐sectional areas found that a lesser proportion (%) of the SMG was composed of duct cells (and thus more acinar cells) in *Slc4a11* ^−/−^ mice (Fig. 7E). Of note, the smaller % ductal area in *Slc4a11* ^−/−^ mice occurred in both female (38% decrease) and male (8.5% decrease) mice (Fig. 7E). These results revealed that *Slc4a11* disruption affected SMG morphology, but not gland weight (Table 3), and resulted in smaller % ductal area, especially in female *Slc4a11* ^−/−^ mice, consistent with the decreased level of NaCl reabsorption (i.e., higher NaCl concentration).

### *Slc4a11* disruption altered *Ascl3* and *Lamc1* mRNA expression in the mouse SMG

Figure 7B shows that there were significant decreases in the percentage of SMG duct cells in *Slc4a11* ^−/−^ mice. We postulated that the changes in ion composition and gland morphology could be due to differential changes in gene expression to compensate for the loss of *Slc4a11* expression in female and male mice. Consequently, qPCR was performed to determine whether *Slc4a11* disruption altered the expression of a panel of nine key genes involved in ion transport and 16 selected genes thought to be involved in the development of the salivary gland cell phenotype/morphology (Table 2, references provided for gene selection rationale). The mRNA levels for members of the ENaC/Degenerin superfamily (*Scnn1a, b* and *g*), Na^+^/H^+^ exchangers (*Slc9a1, a2* and *a3*), water channels (*Aqp5* and *Aqp11*), and the *Cftr* anion channel were comparable in the SMG of *Slc4a11^+/+^* and *Slc4a11* ^−/−^ mice (Table 5). In contrast, for the gland phenotype/morphology‐related genes, we found that mRNA level for *Ascl3*, a transcription factor expressed in a population of salivary gland duct progenitor cells (Arany et al. 2011), was significantly decreased, whereas the expression of *Lamc1*, which regulates acinar cell differentiation (Hoffman et al. 1996), was increased in the Slc4a11^−/−^ mice (Fig. 7F, *P* < 0.01 and *P* < 0.05, respectively). The expression of the other 14 phenotype/morphology‐related genes did not change in the SMG of *Slc4a11* ^−/−^ mice (Table 5). Taken together, the increase in NaCl reabsorption that occurred in the *Slc4a11* ^−/−^ mice may be associated with Ascl3 and/or Lamc1 expression‐related morphological alterations in the SMG. Note that disruption of *Slc4a11* had only a subtle effect at the cellular level on the response to extracellular pH changes and it did not cause a significant change in the mRNA expression of the duct cell markers *Cftr *and *ENaC*. However, there are fewer duct cells in the submandibular glands of *Slc4a11* mice suggesting that the remaining duct cells may have attempted to compensate for the decrease in duct cell numbers.

**Table 5:** mRNA expression of genes as determined by qPCR analysis.

Gene	*Slc4a11^+/+^*	*n*	*Slc4a11* ^−/−^	*n*
*Slc9a1*	62.58 ± 10.40	6	78.31 ± 28.16	6
*Slc9a2*	4.34 ± 0.42	6	3.66 ± 0.72	6
*Slc9a3*	4.98 ± 0.56	6	3.80 ± 1.24	6
*Scnn1a*	24.90 ± 3.29	6	34.13 ± 13.25	6
*Scnn1b*	44.32 ± 9.37	6	28.97 ± 5.11	6
*Scnn1g*	9.80 ± 1.30	6	7.32 ± 1.67	6
*Cftr*	5.68 ± 0.96	6	9.37 ± 3.36	6
*Aqp5*	9849.58 ± 2719.31	6	10080.50 ± 2576.08	6
*Aqp11*	373.23 ± 234.50	6	34.78 ± 28.82	6
*Ascl3*	2596.10 ± 210.39	6	**1138.84** ± **302.64** **	6
*Cdh1*	8889.45 ± 2244.95	6	8899.72 ± 1215.83	6
*E2f1*	155.54 ± 75.74	6	135.06 ± 79.92	6
*Eda*	348.65 ± 169.05	6	256.49 ± 91.85	6
*Edar*	45.92 ± 8.62	6	65.26 ± 31.60	6
*Eda2r*	3928.86 ± 645.63	6	3617.77 ± 830.71	6
*Edaradd*	254.62 ± 74.43	6	699.81 ± 448.38	6
*Gjb1*	5350.51 ± 1354.60	6	5006.50 ± 1447.00	6
*Itga3*	561.52 ± 122.74	6	1049.28 ± 453.96	6
*Itgb1*	5066.08 ± 1095.85	6	4448.26 ± 926.47	6
*Lama1*	294.42 ± 238.82	6	95.18 ± 76.63	6
*Lamb1*	1286.36 ± 435.62	6	1042.43 ± 150.92	6
*Lamc1*	263.87 ± 41.15	6	**1267.66** ± **307.85** *	6
*P2ry1*	593.05 ± 331.51	6	411.73 ± 254.41	6
*Tgfb2*	3157.31 ± 1166.47	6	1275.61 ± 433.55	6
*Tgfb3*	430.21 ± 74.72	6	513.04 ± 129.55	6

Values are the means ± SEM, *n* represents the number of glands used in each qPCR experiment. *P < 0.05, **P < 0.01 in bold, versus glands of *Slc4a11^+/+^* mice.

## Discussion

SLC4A11 is expressed in many tissues including epithelial tissues (Parker et al. 2001; Park et al. 2004; Damkier et al. 2007). *Slc4a11* showed the highest level of mRNA expression of the *Slc4* gene family members in the murine corneal endothelium (Shei et al. 2013), consistent with our transcriptional data in which *Slc4a11* expression in the mouse salivary glands was dramatically higher (at least eightfold greater) than the other *Slc4* family members (Gao et al. 2018). Previous studies showed that the Slc4 members display basolateral and/or apical membrane distributions in different tissues (Park et al. 2002; Endo et al. 2006; Fukuda et al. 2013; Gholami et al. 2014; Gildea et al. 2015; Namkoong et al. 2015), whereas Slc4a11 has been reported to be localized to the basolateral membrane of acinar and duct cells in the rat SMG (Park et al. 2004). However, our results in mice indicated that Slc4a11 has a diffuse intracellular expression in both the acinar and duct cells of the mouse SMG. Thus, it seems likely that Slc4a11 expression and localization are species‐specific. Some of this variability could also be explained by different Slc4a11 variants. Indeed, Kao et al. (2016) reported that SLC4A11‐A was expressed in the intracellular compartment of the human corneal endothelium, whereas the SLC4A11‐B and SLC4A11‐C variants were plasma membrane proteins. The diffuse pattern of the Slc4a11‐specific staining in the salivary glands does not appear to correspond to a specific intracellular compartment. However, the large secretory granules in the apical region of the granular duct in Figure 2 (panel A) are devoid of Slc4a11‐specific staining, demonstrating that not all intracellular compartments contain Slc4a11. Given that the antibody used in this study recognizes the water‐soluble C‐terminal of Slc4a11, one possibility is that the protein has been cleaved and the antibody is detecting something other than the full‐length protein. Moreover, we did not distinguish between the expression of different Slc4a11 variants in the mouse SMG as the isoform annotation is rapidly evolving, but characterization of presence and distribution of Slc4a11 isoforms might help clarify the results we observed in this study. Preliminary *de novo* transcript assembly results from Cufflinks using our RNA‐seq data (Trapnell et al. 2012) suggested that Slc4a11 has three isoforms in male mouse SMG, but only one in female SMG (data not shown). However, we did not perform differential analysis of their expression levels since short reads‐based isoform expression comparison is generally not considered accurate and would require a long‐read sequencing strategy rather than what was previously used (Gao et al. 2018).

The physiological role of Slc4a11 is not well understood. Most previous studies have focused on the link between *SLC4A11* mutations and human corneal disorders (Vilas et al. 2012; Kim et al. 2015; Kumawat et al. 2016; Hand et al. 2017). Human SLC4A11 has been reported to mediate water movement uncoupled from solute flux, similar to AQP proteins, which might explain the human corneal diseases caused by *SLC4A11* mutations (Vilas et al. 2013; Soumittra et al. 2014). In *Slc4a11* knockout mice, sodium chloride crystals form in the cornea and polyuria is associated with an increased loss of NaCl and hypo‐osmolarity of the urine (Groger et al. 2010; Han et al. 2013). Gröger et al. (2010) and Han et al. 2013 found that *Slc4a11* disruption caused decreased urine osmolarity and lower urine electrolyte concentrations in the knock‐out mice. Overall, these studies suggest that Slc4a11 may be involved in water movement and ion homeostasis. Of note, the *Slc4a11* null mouse strain used in this study does not have the exact corneal pathology of CHED (Lopez et al. 2009). Nevertheless, these mice clearly lack Slc4a11 expression in salivary glands (Fig. 2; and qPCR did not detect Slc4a11 mRNA in the salivary glands of *Slc4a11* null mice, data not shown). Consequently, the lack of CHED corneal pathology most likely has no bearing on the salivary gland findings in this paper. It is possible based on the results of this study that patients with CHED, Haroyan syndrome, and FECD due to *SLC4A11* mutations could present with changes in submandibular function regarding fluid secretion and potentially sex differences could also be present. Such patients could be tested clinically for a submandibular gland phenotype.

Given the high expression of Slc4a11 in both acinar and duct cells of the mouse SMG, we predicted that Slc4a11 might also be involved in fluid and/or ion secretion in the mouse SMG. The current salivary gland secretion model states that the acinar cells secrete most of the fluid and that the ducts subsequently reabsorb NaCl in the SMG (Proctor 2016). However, the kinetics and magnitude of fluid secretion were essentially identical in wild‐type and *Slc4a11* null mice suggesting that it is not involved in water movement. In contrast, increased saliva Na^+^ and Cl^−^ concentrations were observed in the SMG of *Slc4a11* knockout mice, especially in the SMG of female *Slc4a11* ^−/−^ mice. NaCl reabsorption and KHCO_3_ secretion occur in salivary gland ducts in a *β*‐adrenergic‐dependent fashion (Denniss et al. 1978). However, the reduced NaCl reabsorption in the SMG of *Slc4a11* null mice occurred in the absence the *β*‐adrenergic receptor agonist isoproterenol (cholinergic receptor agonist CCh only), suggesting that the observed changes induced by *Slc4a11* disruption were independent of *β*‐adrenergic receptor activation. Taken together, these data demonstrated that the NaCl reabsorption defect in the *Slc4a11* null mice occurs in a *β*‐adrenergic‐independent manner.

SLC4A11 is approximately 14–20% identical to the other *SLC4* gene family members, and shares less than 30% protein sequence identity in its transmembrane domain, compatible with the finding that its transport properties are unique within the SLC4 family (Romero et al. 2013; Parker and Boron 2013). Indeed, unlike all other SLC4 members, human SLC4A11 is not involved in HCO_3_^−^ transport (Ogando et al. 2013; Kao et al. 2015; Loganathan et al. 2016), and this study confirmed that heterologous expressed mouse Slc4a11 also does not mediate HCO_3_^−^ transport. Moreover, in HEK293 and PS120 cells SLC4A11 was reported to be a Na^+^‐coupled OH^−^(H^+^) transporter (Park et al. 2004; Ogando et al. 2013). Other studies demonstrated that SLC4A11 functioned as an electrogenic H^+^(OH^−^) transporter that is inhibited by EIPA (Kao et al. 2015; 2016). Zhang et al. (2015) reported that human SLC4A11 acts as an NH_3_/2H^+^ cotransporter that is not affected by extracellular Na^+^, K^+^, or Cl^−^ depletion. Similarly, mouse Slc4a11 acted as a selective H^+^/OH^−^ conductive pathway uncoupled with other ions when expressed in *Xenopus oocytes* (Myers et al. 2016). We also found (Fig. 5) that mouse Slc4a11 mediated H^+^/OH^−^ transport that did not appear to be coupled with Na^+^ or Cl^−^. Park et al. (2004) reported that rat Slc4a11‐mediated Na^+^‐dependent acidification was resistant to 10 *µ*mol/L EIPA. In contrast, another study showed that human SlC4A11‐mediated activity was completely blocked by 1 *µ*mol/L EIPA (Ogando et al. 2013). Human H^+^(OH^−^) and ammonia‐stimulated fluxes were significantly decreased by 30 *µ*mol/L EIPA (Kao et al. 2016). Our results demonstrated that mouse Slc4a11‐mediated H^+^/OH^−^ transport was fully inhibited by a high concentration of EIPA (500 *µ*mol/L) but was not sensitive to 10 *µ*mol/L EIPA. Then again, the SITS‐Affi‐Gel binding assay revealed that SLC4A11 had a much lower affinity for H_2_DIDS than Slc4a1 (Vilas et al. 2011), and as previously reported, we found that Slc4a11 does not appear to be inhibited by 100 *µ*mol/L DIDS (Zhang et al. 2015) or 500 *µ*mol/L DIDS (Park et al. 2004). Of note, *Slc4a11* disruption had little effect on the intracellular pH regulation of the SMG acinar or duct cells. Considering the complex intracellular pH regulation machinery in native SMG cells, we cannot rule out that other pH regulatory mechanisms compensated for the loss of *Slc4a11* expression.

It is not clear what led to the altered NaCl reabsorption by the SMG of *Slc4a11* null mice in this study (Figs. 3C and 4C). It is well established that the epithelial Na^+^ channel (ENaC) and the cystic fibrosis transmembrane conductance regulator (CFTR) play key roles in mouse SMG ductal NaCl reabsorption (Catalán et al. 2010). In addition, Na^+^/H^+^ exchangers regulate NaCl reabsorption in the mouse SMG (Park et al. 2001). However, our qPCR results revealed that the expression of ENaC superfamily members, Na^+^/H^+^ exchangers, water channels and the *Cftr* anion channel was not impaired in the *Slc4a11* null mice, suggesting that *Slc4a11* disruption probably affected NaCl reabsorption through another mechanism. Salt reabsorption mainly occurs in the duct cells of the SMG (Proctor 2016; Melvin et al. 2005; Catalán et al. 2009). Morphological analysis found that fewer duct cells were observed in the SMG of *Slc4a11* null mice, which is consistent with the high NaCl concentration (less NaCl reabsorption) in the stimulated saliva. Of note, the change in NaCl concentration was greater in female than in male *Slc4a11* null mice. Consistent with this latter observation, there was a greater than fourfold impact on both the loss of duct cells and decreased NaCl reabsorption in the female SMG. Given the variety of sex‐specific differences in fluid secretion content and morphology of salivary glands (Gao et al. 2018; Mukaibo et al. 2018; 2019) these results are not too surprising although the mechanism remains unclear. Ascl3 (also known as Sgn1) is specifically expressed in the duct cells of all three major salivary glands (Yoshida et al. 2001) and is involved in the development and maintenance of functional salivary glands (Arany et al. 2011), whereas Laminin‐1, encoded by *Lamc*, has been shown to contribute to development of an acinar‐like phenotype in a human submandibular gland cell line (Hoffman et al. 1996). Consistent with our morphological results, *Ascl3* expression was reduced, whereas *Lamc1* expression was increased in the SMG of *Slc4a11* ^−/−^ mice, suggesting that the greater *Slc4a11* disruption‐induced changes in female SMG morphology may ultimately lead to impaired NaCl reabsorption. The changes in *Ascl* and *Lamc1* gene expression might reflect a shift in duct cell versus acinar cell numbers. Alternatively, the changes in their expression could be responsible for the remodeling.

In summary, mouse Slc4a11 mediates H^+^/OH^−^ transport in a Na^+^‐ and Cl^−^‐independent, EIPA‐sensitive and DIDS‐resistant manner. Despite the abundant expression of Slc4a11 in the mouse SMG, Slc4a11 does not seem to contribute significantly to the intracellular pH regulation of SMG acinar or duct cells, but we cannot rule the possibility that it may regulate pH under different physiological or pathophysiological conditions. On the other hand, Slc4a11 is involved in the regulation of the ductal and acinar morphology of the SMG; therefore, the reduced ductal cell area in especially female *Slc4a11* null mice is likely associated with the impaired NaCl reabsorption. Saliva gland acinar cells secrete an isotonic plasma‐like, NaCl‐rich saliva, which subsequently becomes hypotonic as it flows through the salivary gland duct cells (Proctor 2016; Melvin et al. 2005; Catalán et al. 2009), although the clinical implications of an increase in the NaCl concentration of saliva have not been thoroughly investigated. However, the hypotonicity of saliva (low NaCl) is known to be important for taste (Proctor 2016).

## Conflict of Interest

No conflicts of interest, financial or otherwise, are declared by the authors.

## Data Availability

Gene expression data used in this study are deposited in the Gene Expression Omnibus (GEO accession number GSE96747; 9).
